# Gut Dysbiosis and Plasma Trimethylamine Oxide Are Associated with Subclinical Coronary Atherosclerosis in Obese Patients with Metabolic Dysfunction-Associated Steatotic Liver Disease

**DOI:** 10.3390/nu17172759

**Published:** 2025-08-26

**Authors:** Kittiya Islam, Pattida Kongsomboonchoke, Maneerat Chayanupatkul, Monravee Tumkosit, Pairoj Chattranukulchai, Pinidphon Prombutara, Pisit Tangkijvanich

**Affiliations:** 1 Center of Excellence in Hepatitis and Liver Cancer, Department of Biochemistry, Chulalongkorn University, Bangkok 10330, Thailand; kittiya.islam@gmail.com (K.I.); gee.pattida@gmail.com (P.K.); 2 Center of Excellence in Alternative and Complementary Medicine for Gastrointestinal and Liver Diseases, Department of Physiology, Faculty of Medicine, Chulalongkorn University, Bangkok 10330, Thailand; maneeratc@gmail.com; 3 Department of Radiology, Faculty of Medicine, Chulalongkorn University, King Chulalongkorn Memorial Hospital, Bangkok 10330, Thailand; monravee.t@chula.ac.th; 4 Division of Cardiovascular Medicine, Department of Medicine, Faculty of Medicine, Chulalongkorn University, and Cardiac Center, King Chulalongkorn Memorial Hospital, Bangkok 10330, Thailand; pairoj.md@gmail.com; 5 Mod Gut Co., Ltd., Bangkok 10600, Thailand; pinidphon.p@chula.ac.th; 6 Omics Sciences and Bioinformatics Center, Faculty of Science, Chulalongkorn University, Bangkok 10330, Thailand

**Keywords:** atherosclerosis, cardiovascular disease, gut microbiota, obesity, short-chain fatty acids, steatotic liver disease, trimethylamine oxide

## Abstract

**Background/Objectives**: Gut microbiota has been implicated in the pathogenesis of metabolic dysfunction-associated steatotic liver disease (MASLD) and cardiovascular disease (CVD). This study aimed to identify associations between gut dysbiosis and MASLD, regarding body mass index (BMI) and subclinical coronary atherosclerosis (SCA). **Methods**: We conducted a cross-sectional study of 202 patients with MASLD who had no previous history of CVD. The severity of MASLD was evaluated using a magnetic resonance imaging-based method, and SCA was measured by assessing coronary artery calcification (CAC). Gut microbiota was assessed in fecal specimens using sequencing targeting the V4 region of the *16S rRNA* gene. **Results**: Our results demonstrated that gut microbial profiles between low- and high-BMI groups (<30 vs. ≥30 kg/m^2^) differed significantly in beta-diversity, but not in alpha-diversity indices. At the genus level, we identified *Megamonas*, *Sutterella*, *Catenibacterium*, and *Odoribacter*, enriched in the high BMI group. Compared to the low CAC group (<100 AU), MASLD patients with high CAC scores (≥100 AU) exhibited enrichment in *Ruminococcus gnavus*, *Bacteroides*, and *Lachnoclostridium*, along with depletion of several short-chain fatty acid (SCFA)-producing bacteria, such as *Faecalibacterium*. Multivariate analysis demonstrated that older age, the presence of diabetes, high BMI, fibrosis stage F3-F4, and high plasma trimethylamine oxide (TMAO) levels were independently associated with a high CAC score in patients with MASLD. **Conclusions**: These data indicated that gut dysbiosis and related metabolites, in association with advanced liver disease, were potential contributors to the progression of SCA in obese patients with MASLD.

## 1. Introduction

Metabolic dysfunction-associated steatotic liver disease (MASLD), previously termed non-alcoholic fatty liver disease (NAFLD), has been recognized as one of the most common chronic liver diseases, affecting approximately 30–35% of the global adult population [1]. MASLD is mainly characterized by a combination of steatotic liver disease and phenotypic features of cardiometabolic risk factors [1]. Regarding hepatic manifestations, MASLD can lead to advanced fibrosis, cirrhosis, and hepatocellular carcinoma (HCC) [2]. As a high proportion of patients with MASLD will ultimately develop advanced liver disease, it is necessary to assess the severity of liver disease in managing patients with MASLD. Currently, liver biopsy is the gold standard for determining the extent of steatosis and fibrosis. However, this procedure is invasive, has potential complications, and is prone to sampling errors [3]. As a result, non-invasive methods such as magnetic resonance imaging-proton density fat fraction (MRI-PDFF) and magnetic resonance elastography (MRE) are now considered the best options for evaluating steatosis and fibrosis, respectively [4].

In addition to liver-related complications, accumulating data demonstrate that MASLD is strongly linked to an increased risk of extrahepatic disorders, such as cancers, chronic kidney disease (CKD), and cardiovascular disease (CVD). Indeed, CVD, particularly coronary artery disease, is a significant cause of morbidity and mortality in patients with MASLD [5]. Subclinical coronary atherosclerosis (SCA), an early indicator of atherosclerotic burden, is defined by coronary arterial plaques with or without thrombosis in the absence of overt clinical manifestations [6]. A recent meta-analysis has indicated that MASLD is significantly associated with SCA, particularly among patients with advanced fibrosis [7]. Indeed, MASLD is also closely associated with type 2 diabetes mellitus (T2DM) and obesity. According to the Asian-Pacific guideline, persons with body mass index (BMI) of 18.5–22.9, 23–24.9, 25–29.9, and ≥30 kg/m^2^ were classified as lean, overweight, obese class I, and obese class II, respectively [8]. However, approximately 10–20% of Asian individuals with MASLD are classified as having normal BMI [9]. A recent meta-analysis has emphasized the weight-dependent effect, as obese MASLD individuals are significantly more metabolically unhealthy than those with normal BMI [10]. Although BMI may have some shortcomings, including its inability to differentiate body composition, it provides a good indicator for both sexes and all age groups within the adult population. Given the close connection among obesity, MASLD, and CVD risk, it is necessary to detect SCA early and understand the mechanisms that interact among these cardiometabolic diseases beyond traditional risk factors.

Emerging evidence suggests that gut microbiota may influence the development and severity of MASLD through the gut–liver axis [11,12]. Notably, gut dysbiosis is characterized by an imbalance in the microbial community, with an increased abundance of potentially harmful bacterial taxa and a reduced abundance of beneficial bacterial taxa, which contributes to the pathogenesis of MASLD. An altered intestinal barrier and translocation of bacterial components and metabolites, including lipopolysaccharides (LPS) and other endotoxins, could induce systemic inflammation and potentially drive MASLD progression [13]. Likewise, several reports have demonstrated that alterations in gut microbial composition and increased intestinal permeability, or leaky gut, may play a crucial role in the development of clinical CVD [14]. Additionally, gut dysbiosis affects CVD by producing related metabolites, particularly trimethylamine oxide (TMAO). This metabolite is produced by converting choline, betaine, and other precursor compounds into trimethylamine (TMA) by gut bacteria, which is then oxidized to TMAO in the liver [15]. TMAO can accelerate atherosclerosis through several mechanisms, including modulating lipid metabolism, increasing reactive oxygen species (ROS) production, promoting platelet aggregation and vascular inflammation, and ultimately leading to the formation of atherogenic plaques [16]. However, current information linking gut dysbiosis to subclinical coronary atherosclerosis in patients with MASLD remains inadequate and thus needs further investigation.

In this cross-sectional study, we aimed to investigate the diversity and composition of gut microbiota in a well-characterized cohort of Thai individuals with MASLD, focusing on BMI and SCA. In this respect, we used MRE and MRI-PDFF measurements to evaluate the severity of fibrosis and steatosis, respectively. To quantify subclinical coronary atherosclerosis, we employed coronary artery calcification (CAC), a reliable and non-invasive tool for assessing the severity of CVD in asymptomatic individuals [17]. It has been shown that CAC is closely linked to the severity of atherosclerosis and its prognosis, irrespective of conventional CVD risk factors [17]. Moreover, we identified various clinical and laboratory parameters that may be independently associated with the severity of CAC using logistic regression analyses. Together, our findings might provide additional valuable data to better understand how gut dysbiosis affects the pathogenesis of MASLD and SCA.

## 2. Materials and Methods

### 2.1. Participants and Study Design

The participants in this report were selected from a subgroup of Thai patients aged ≥18 years, who were undergoing follow-up in the outpatient clinic in the King Chulalongkorn Memorial Hospital in Bangkok, Thailand. These patients had evidence of liver steatosis based on vibration-controlled transient elastography (FibroScan with controlled attenuation parameter, CAP > 248 dB/m) or findings on ultrasound examination. Among them, we further confirmed the diagnosis of liver steatosis by using MRI-PDFF (MRI-PDFF ≥ 5.4%) [18], and there were 202 patients enrolled in this study. Exclusion criteria were (1) associated with other chronic liver diseases, such as chronic viral hepatitis (HBsAg, anti-HCV positive), autoimmune hepatitis (anti-nuclear antibodies, anti-smooth muscle antibodies positive), and hemochromatosis (high transferrin saturation and ferritin levels); (2) human immunodeficiency virus (HIV) infection; (3) the presence of decompensated cirrhosis and complications, including ascites, variceal bleeding, and HCC, (4) self-report of excessive alcohol intake (>10 g/day in women and >20 g/day in men), and (5) previous history of CVD such as coronary artery disease or stroke.

The patients were advised to avoid herbal or nutritional supplements, proton pump inhibitors, antibiotics, prebiotics, and probiotics for at least 4 weeks before recruitment. Clinical data and anthropometric measurements were documented at enrollment. The protocols (IRB No. 981-64 and No. 769/66) were approved by the Institutional Review Board of the Faculty of Medicine, Chulalongkorn University, Bangkok, Thailand. The study adhered to the Declaration of Helsinki and the principles of good clinical practice, following the provision of written informed consent by the patients.

### 2.2. Liver Stiffness and Steatosis Measurement

MRE and MRI-PDFF were used to assess liver stiffness and steatosis by the MR imaging system Philips Ingenia at 3.0 T (Philips Healthcare, Best, The Netherlands). According to a systematic review and meta-analysis in MASLD, the cut-off values for fibrosis stages ≥F1, ≥F2, ≥F3, and F4 by MRE measurement were 2.6, 3.0, 3.6, and 4.7 kPa, respectively [4]. As to MRI-PDFF, the cut-off points for steatosis grades ≥1, ≥2, and ≥3 were 5.4%, 15.4%, and 20.4%, respectively [18]. A radiologist evaluated and interpreted the acquired imaging information without being aware of the participant’s clinical and laboratory data.

### 2.3. CAC Quantification Using a Multisection CT Scan

Participants underwent CAC quantification using a multisection CT scan (Somatom Sensation 64; Siemens Medical Systems), as described previously [19]. This process was used to quantify coronary calcium by multiplying the weighted density score by the pixel area of the calcification speck [20]. The standard scan parameters consisted of a 3 mm section thickness, 1.2 × 24 mm collimation, a 0.37 s rotation, and spiral mode with 120 kVp at 80 mAs, with reconstruction operating at 60% of the R-R interval. The Agatston scores assessing the severity of CAC were confirmed by a single experienced observer blinded to the patient’s clinical and laboratory data. The presence of SCA is established by a CAC score of >0 Agatston units (AU), while a CAC score of ≥100 and ≥400 AU characterizes intermediate and severe risk, respectively. In this study, 97 (48%) patients were evaluated for the CAC score.

### 2.4. Fecal Collection, DNA Extraction, and Sequencing

Fecal samples collected from the participants in the DNA/RNA Shield™-Fecal Collection tubes (Zymo Research Corp, Irvine, CA, USA) were shaken and stored at −80 °C until further analysis. The collected specimens were then extracted for DNA by the ZymoBIOMICS™ DNA Miniprep Kit (Zymo Research Corp, Irvine, CA, USA), following the manufacturer’s instructions. The purity and concentration of total DNA were then measured using a DeNovix™ UV-Vis spectrophotometer (DeNovix Inc., Wilmington, DE, USA), and the DNA was stored at −20 °C. Subsequently, amplicon-based 16S rRNA gene sequencing located in the V4 hypervariable regions was amplified based on forward primer 515F (5′-GTGCCAGCMGCCGCGGTAA-3′) and reverse primer 806R (5′-GGACTACHVGGGTWTCTAAT-3′). Additionally, paired-end sequencing was conducted on the Illumina MiSeq 2 × 300 bp platform (San Diego, CA, USA) at Mod Gut Co., Ltd. (Bangkok, Thailand).

### 2.5. Gut Microbiota Data Processing and Analysis

The gut microbiome analysis was examined using QIIME 2 48 (version 2024.2) as the raw sequence data was demultiplexed using the q2-demux plugin. Next, reads with expected errors (maxEE) higher than 3.0 were discarded using denoising software DADA2 (via q2-dada2), and the mitochondria and chloroplast 16S sequences were then eliminated. A phylogeny was constructed from representative sequences using the align_to_tree_mafft_fasttree command of the q2-phylogeny plugin. Alpha-diversity indexes and beta-diversity metrics were assessed using the q2-diversity plugin after rarefying the samples to 12,000 reads. Next, Principal Coordinate Analysis (PCoA) was analyzed on the beta-diversity distance metrics. Permutational Multivariate Analysis of Variance (PERMANOVA) was conducted to assess differences in microbial community composition between groups. Statistical significance was defined as *p* < 0.05. The amplicon sequence variants (ASVs) were taxonomically assigned using the classify-sklearn naive Bayes taxonomy classifier against the Silva (version 138) 99% reference sequences. The Spearman Rank Coefficient was applied to assess the relation between clinical parameters and microbial genera. The clustermap function from the seaborn library in Python version 3.10.13 was employed to yield a correlation graph representing clusters.

### 2.6. Fecal BCoAT Gene Assessment

The butyryl-CoA: acetate CoA transferase (BCoAT) was assessed to estimate the concentration of butyrate in fecal specimens by all butyrate-producing bacteria. Briefly, the quantification was tested by using qPCR based on 4X CAPITAL™ qPCR Green Master Mix (Biotechrabbit GmbH, Berlin, Germany) with the following degenerate primers: (forward primer) 5′-GCIGAICATTTCACITGGAAYWSITGGCAYATG-3′ and (reverse primer) 5′-CCTGCCTTTGCAATRTCIACRAANGC-3′ as previously described [21]. The qPCR conditions began with a DNA-denaturation step at 95 °C for 15 min, followed by 40 cycles of denaturation at 95 °C for 15 s, annealing at a primer-specific temperature for 20 s, and extension at 72 °C for 30 s. The quantification of the *BCoAT* gene was examined by normalizing its assay using the *V3-V4* gene as the representative of total bacteria.

### 2.7. Plasma Biomarker Measurement

Peripheral blood samples were collected from the patients, handled within two hours to separate the plasma component, and preserved at −80 °C until analysis. Plasma intestinal fatty acid binding protein (I-FABP), a biomarker of intestinal permeability [22], was determined using an ELISA kit (Hycult Biotech, Uden, The Netherlands) after being diluted at 1:2, following the manufacturer’s protocol. Plasma lipopolysaccharide-binding protein (LBP) concentrations, an accurate biomarker of LPS and endotoxemia [23], were analyzed using ELISA kits (Hycult Biotech, Uden, The Netherlands) after being diluted at 1:1000. Plasma choline, betaine, and TMAO levels were assessed using ultra-high performance liquid chromatography-tandem spectrometry (UHPLC-MS/MS), as previously described [24]. In this study, the limits of detection were 47 pg/mL for I-FABP, 0.94 pg/mL for LPS, and 0.05 µM for TMAO. The intra-assay coefficients of variation (CVs) ranged from 3.2 to 6.4% for I-FABP, 4.13 to 6.7% for LPS, and 1.40 to 7.62% for TMAO, whereas the inter-assay CVs of the respective tests ranged from 0.2 to 1.6%, 3.13 to 4.62%, and 1.65 to 7.15%, respectively.

### 2.8. Statistical Analysis

The statistical evaluation of the variables was performed using SPSS (version 22.0.0, SPSS Inc., Chicago, IL, USA) and GraphPad Prism (version 9.5.0, GraphPad Software, Inc., San Diego, CA, USA) as appropriate. Categorical data were analyzed using the Chi-square test and one-way ANOVA. The Mann–Whitney test was applied to compare the distributions of two unmatched groups. Spearman’s rank test was applied to analyze correlations between parameters. Univariate and multivariable analyses were performed using SPSS binary logistic regression to identify the parameters associated with a CAC greater than 100. A *p*-value < 0.05 was considered statistically significant.

## 3. Results

### 3.1. Clinical Characteristics of Patients

The clinical characteristics of the patients in this study, categorized by BMI (<30 kg/m^2^, *n* = 151 and ≥30 kg/m^2^, *n* = 51), are presented in Table 1. Compared to patients with low BMI, the high BMI group was significantly younger but had a significantly higher MRI-PDFF value and average CAC score. There was no difference in gender distribution, as well as in the frequencies of T2DM, hypertension (HT), and dyslipidemia (DLP) between the groups. Additionally, the biochemical blood tests, including liver function and renal function tests, as well as the average MRE measurement, were similar between groups.

### 3.2. The Alpha and Beta Diversities of Gut Microbiota

**Figure 1:**
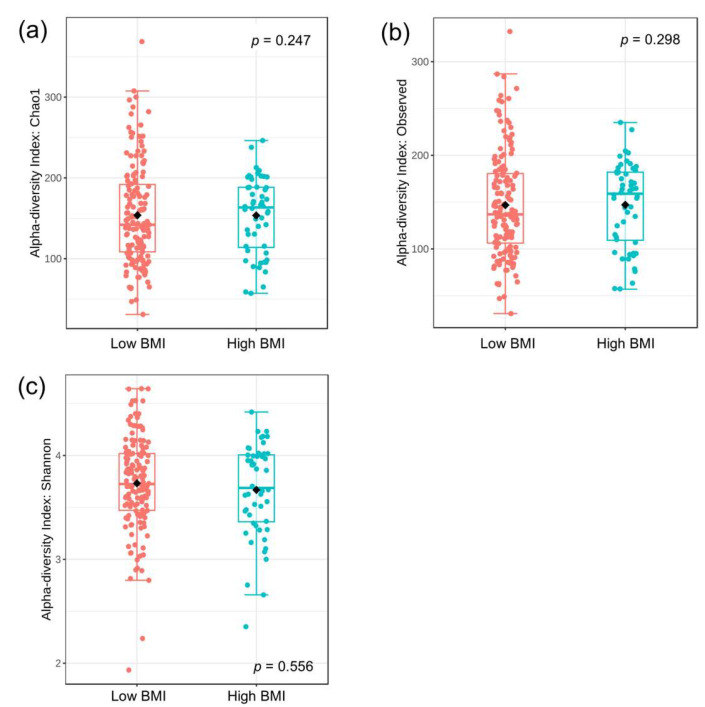
Alpha diversities between the low and high BMI groups: (**a**) Chao1 index, (**b**) observed index, and (**c**) Shannon index. Statistical measures were median, interquartile range, and outliers. The statistical analysis was performed using the Mann–Whitney test.

The alpha diversities of bacteria, as measured by the Chao1, Observed, and Shannon indices, were compared between patients in low- and high-BMI categories (<30 vs. ≥30 kg/m^2^). Our results demonstrated that there were no significant differences in any of the indices between the two studied groups (Mann–Whitney test, *p* = 0.247, 0.298, and 0.556, respectively). These results indicated that bacterial richness and evenness did not differ according to the patients’ BMI (Figure 1a–c).

**Figure 2:**
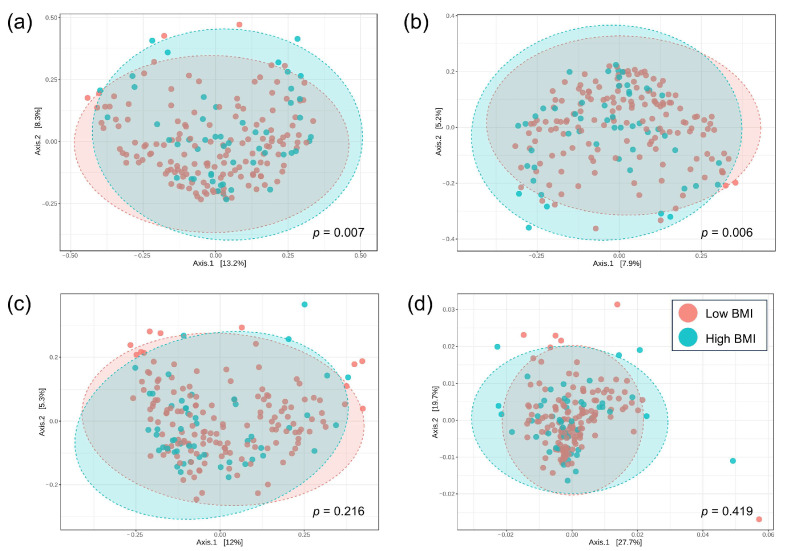
The principal coordinate analysis (PCoA) of beta-diversity profiling between the low and high BMI groups: (**a**) Bray–Curtis index, (**b**) Jaccard index, (**c**) Unweighted Unifrac index, (**d**) Weighted Unifrac index. The differences in beta diversity were tested PERMANOVA.

To measure the compositional dissimilarity of bacteria between the low and high BMI groups, the Bray–Curtis, Jaccard, Unweighted Unifrac, and Weighted Unifrac indices were applied in conjunction with PERMANOVA tests. The results were then visualized in a Principal Coordinate Analysis (PCoA) plot, as shown in Figure 2a–d. The data demonstrated a significant difference in beta diversity between the low and high BMI groups, as indicated by the Bray–Curtis and Jaccard indices (PERMANOVA, *p* = 0.007 and *p* = 0.006, respectively). However, there was no such difference regarding Unweighted Unifrac and Weighted Unifrac indices (*p* = 0.216 and *p* = 0.419, respectively).

We also compared the diversities of gut microbiota in association with CAC scores (<100 vs. ≥100 AU). Regarding alpha diversities, only the Shannon index showed a significant difference between groups (Mann–Whitney test, *p* = 0.025). However, the Chao1, Observed, and Simpson indices did not differ significantly (*p* = 0.103, *p* = 0.087, and *p* = 0.066, respectively) (Figure S1). For beta diversities, only the Bray–Curtis displayed a significant difference between the low and high CAC score groups (PERMANOVA, *p* = 0.049). In contrast, no significant difference was found for Jaccard, Unweighted Unifrac, and Weighted Unifrac indices (*p* = 0.065, *p* = 0.120, and *p* = 0.387, respectively) (Figure S2).

### 3.3. Gut Bacterial Compositional Analysis

**Figure 3:**
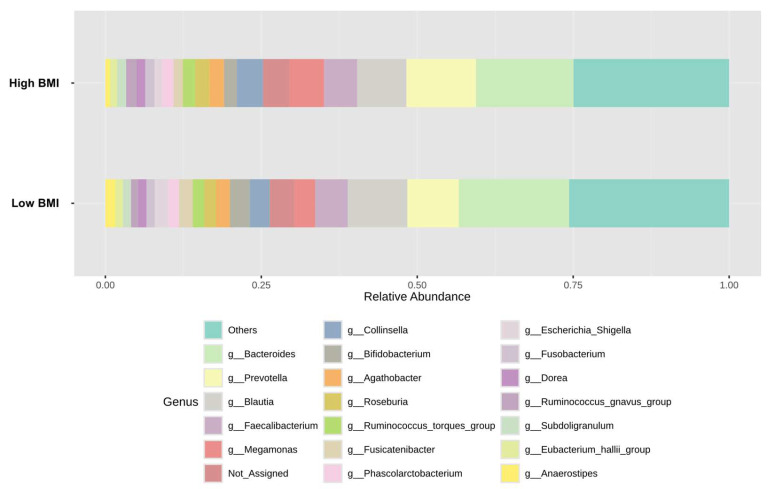
Top 20 of relative bacterial composition at the genus level of the low and high BMI groups.

To determine whether the difference in gut microbial composition was associated with obesity, we compared the top 20 relative abundances of bacteria at the genus level according to patients’ BMI category (Figure 3 and Table S1).

**Figure 4:**
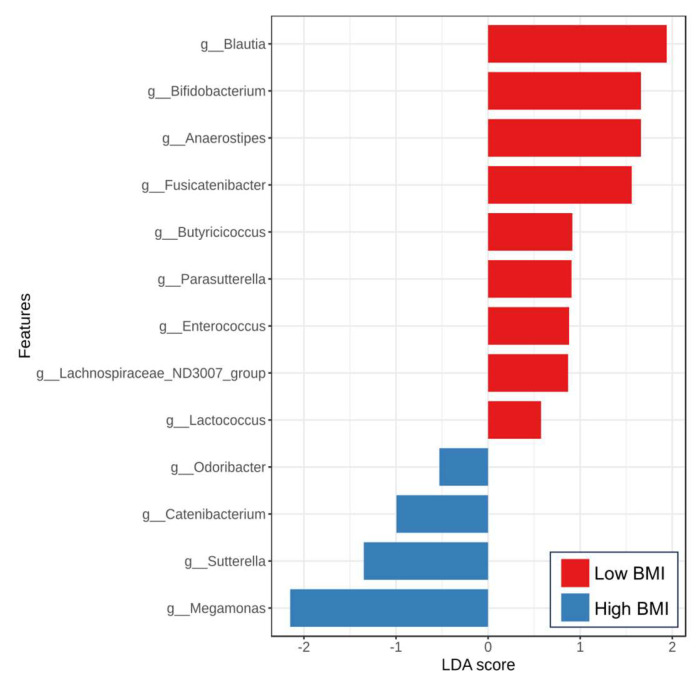
Linear discriminant analysis (LDA) effect size (LEfSe) analysis of gut microbiota between the low and high BMI groups at the genus level (LAD > 2, *p* < 0.05).

To further evaluate the significant differences in microbiota composition between low-and high-BMI groups, the LEfSe method was performed (Figure 4). Our results showed that the enriched *Blautia*, *Bifidobacterium*, *Anaerostipes*, and *Butyricicoccus*, among others, were observed in patients with low BMI. Additionally, several bacterial genera, including *Megamonas*, *Sutterella*, *Catenibacterium*, and *Odoribacter*, were found to be increased in the high BMI subjects. These data indicated a significant distinction in gut microbiota composition among patients with MASLD based on the severity of obesity.

**Figure 5:**
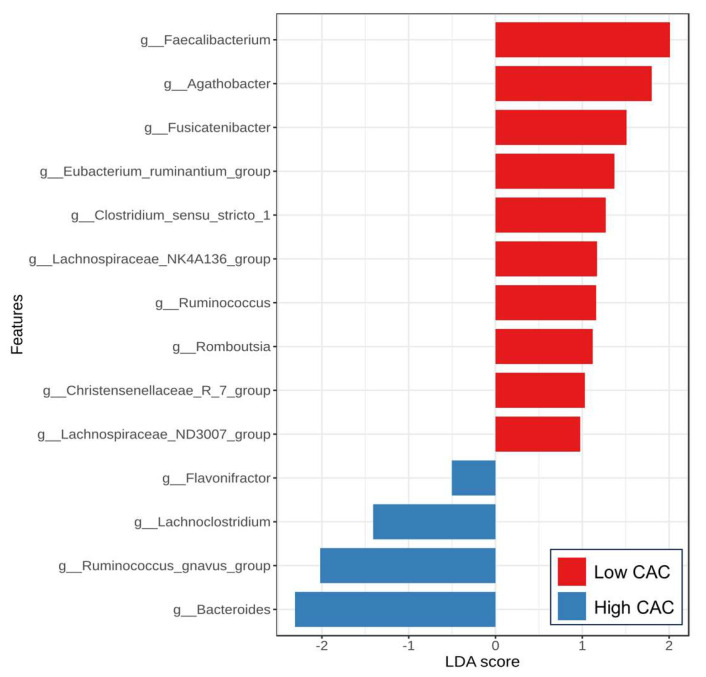
Linear discriminant analysis (LDA) effect size (LEfSe) analysis of gut microbiota between the low and high CAC score groups at the genus level (LAD > 2, *p* < 0.05).

Regarding CAC severity, the analysis using the LEfSe method also demonstrated the discriminating bacterial abundance between groups (Figure 5). Among the low CAC group, the enrichment of short-chain fatty acid (SCFA)-producing bacteria, including *Faecalibacterium*, *Agathobacter*, *Fusicatenibacter*, *Eubacterium*, *Lachnospiraceae*, and *Christensenella*, was detected. Among patients with high CAC scores above 100 AU, an increased abundance of *Ruminococcus gnavus*, *Bacteroides*, and *Lachnoclostridium* was observed compared to those with less severe CAC scores. Notably, the CAC score was positively correlated with the relative abundance of *Ruminococcus gnavus* (r = 0.232, *p* = 0.022) and *Bacteroides* (r = 0.221, *p =* 0.029). In contrast, the CAC score was negatively correlated with the abundance of several genera, including *Faecalibacterium* (r = −0.345, *p* = 0.001), *Agathobacter* (r = −0.252, *p* = 0.012), *Fusicatenibacter* (r = −0.408, *p* < 0.001), and *Lachnospira* (r = −0.289, *p* = 0.004).

### 3.4. Fecal BCoAT Level

We also measured the levels of BCoAT in fecal specimens to evaluate the production of butyrate by gut microbiota. The results showed no difference in BCoAT expression between the low and high BMI groups (0.030 ± 0.040 vs. 0.035 ± 0.063, *p* = 0.539). However, patients with a high CAC score (≥100 AU) had significantly lower fecal BCoAT levels than those with a low CAC score <100 AU (0.020 ± 0.024 vs. 0.041 ± 0.068, *p =* 0.046). Fecal BCoAT had a weak negative correlation with age (r = −0.177, *p* = 0.012), MRE (r = −0.153, *p* = 0.031), and CAC score (r = −0.265, *p* = 0.008), but displayed no correlation with PDFF (r = 0.041, *p* = 0.571).

### 3.5. Plasma Biomarker Levels

Plasma concentrations of I-FABP and LBP, representing gut epithelial permeability and bacterial translocation, respectively, were further evaluated. Our results demonstrated that the low BMI group had similar I-FABP levels compared with the high BMI group (778.5 ± 887.3 vs. 648.2 ± 611.1 ng/mL, *p* = 0.337) but showed significantly lower LBP levels (15,174.8 ± 6449.8 vs. 18,577.5 ± 7363.7 ng/mL, *p* = 0.002). Comparing groups regarding CAC scores, there was a significant increase in I-FABP levels in patients with high CAC compared to those with low CAC scores (1137.0 ± 1306.8 vs. 560.0 ± 445.4 ng/mL, *p* = 0.002). Regarding LBP, the high CAC group tended to have higher levels than the low CAC group, but no significant difference was found (17,654.8 ± 8053.6 vs. 15,688.6 ± 6450.0 ng/mL, *p* = 0.187).

Plasma TMAO and related biomarkers were further investigated. Comparing the low and high BMI groups, there was no significant difference regarding choline (13.1 ± 6.4 vs. 13.6 ± 5.1 µM, *p* = 0.641), betaine (41.3 ± 26.4 vs. 41.4 ± 25.1 µM, *p* = 0.983), and TMAO levels (4.1 ± 7.5 vs. 3.0 ± 2.6 µM, *p* = 0.339). Comparing the low and high CAC groups, there was no significant difference regarding choline (12.6 ± 5.8 vs. 14.5 ± 7.6 µM, *p =* 0.177) and betaine (35.1 ± 25.9 vs. 39.8 ± 26.4 µM, *p* = 0.381). However, patients with a high CAC score had significantly elevated TMAO levels than those with a low CAC score (6.0 ± 8.0 vs. 2.2 ± 2.1 µM, *p* = 0.002).

**Figure 6:**
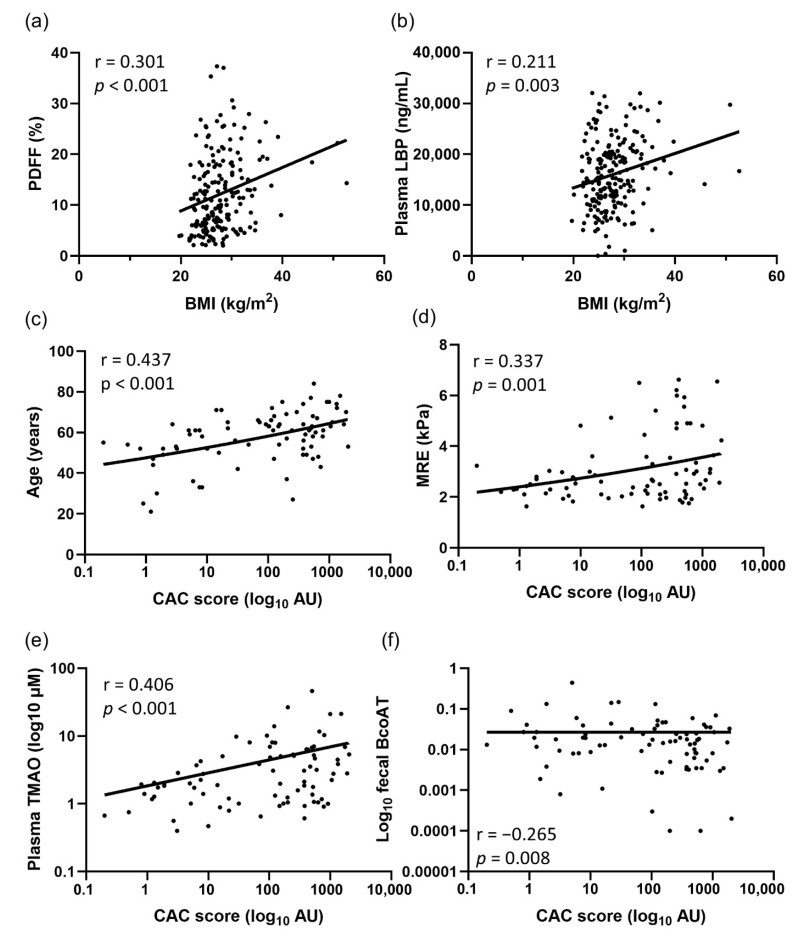
The correlations of selected parameters regarding BMI and CAC scores: (**a**) BMI vs. PDFF, (**b**) BMI vs. LBP, (**c**) CAC score vs. age, (**d**) CAC score vs. MRE, (**e**) CAC score vs. TMAO, and (**f**) CAC score vs. BCoAT. The statistical analysis was performed using Spearman’s rank test.

Plasma I-FABP was significantly correlated with patients’ age (r = 0.338, *p* < 0.001), LBP (r = 0.202, *p* = 0.004), MRE (r = 0.395, *p* < 0.001), and CAC score (r = 0.347, *p* < 0.001), but showed a negative correlation with fecal BCoAT (r = −0.234, *p* = 0.001). Plasma LBP level showed a positive correlation with BMI (r = 0.211, *p* = 0.003) and CAC score (r = 0.405, *p* < 0.001) but displayed a negative correlation with fecal BCoAT (r = −0.268, *p* < 0.001). The significant correlations of selected parameters are shown in Figure 6.

Plasma biomarkers in correlation with the abundance of bacterial genera were also analyzed. TMAO levels showed a weakly positive correlation with Bacteroides (r = 0.145, *p* = 0.040) and *Escherichia_Shigella* (r = 0.157, *p* = 0.025). However, they exhibited a negative correlation with the abundance of several SCFA-producing bacteria, including Faecalibacterium (r = −0.321, *p* < 0.001), Agathobacter (r = −0.322, *p* < 0.001), Fusicatenibacter (r = −0.217, *p* = 0.002), Eubacterium (r = −0.237, *p* = 0.001), and Lachnospiraceae (r = −0.211, *p* = 0.003). Additionally, plasma I-FABP concentrations were positively correlated with *Bacteroides* (r = 0.147, *p* = 0.039) and *Escherichia_Shigella* (r = 0.172, *p* = 0.015) but negatively correlated with *Faecalibacterium* (r = −0.161, *p* = 0.024). For LBP, the marker was negatively correlated with the abundance of Blautia (r = −0.163, *p* = 0.021).

### 3.6. Univariate and Multivariate Analyses

We further examined whether any of the parameters studied were independently associated with a high CAC score greater than 100 AU. The variables entered into univariate and multivariate analyses included age, gender, BMI, T2DM, HT, DLP, smoking, serum aspartate aminotransferase (AST), alanine aminotransferase (ALT), serum albumin, platelet count, estimated glomerular filtration rate (eGFR) liver steatosis, and liver fibrosis, as well as biomarkers associated with gut dysbiosis, including fecal BCoAT, plasma I-FABP, LBP, choline, betaine and TMAO. Multivariate analysis demonstrated that older age, the presence of T2DM, high BMI, Fibrosis stage F3-F4, and high plasma TMAO levels were independently associated with a high CAC score in patients with MASLD (Table 2).

## 4. Discussion

MASLD is currently the most common chronic liver disease associated with several systemic metabolic disorders, particularly CVD consequences [1,5]. MASLD has become an increasing public health concern in Asian populations due to the obesity epidemic related to Westernized dietary patterns and lifestyle changes. A meta-analysis has demonstrated that the severity of metabolic dysregulation is weight-dependent, and obese individuals typically display more metabolic dysfunction compared with non-obese patients [10]. Additionally, obese individuals with MASLD commonly exhibit worse clinical outcomes, including end-stage liver disease and extrahepatic complications, compared to those with normal weight [25]. In this study, we also demonstrated that MASLD with advanced fibrosis/cirrhosis (F3-F4) was independently associated with high CAC scores, aligning with the findings in a recent report from eastern China [26]. These results are also similar to those of the meta-analysis in MASLD, indicating an association between fibrosis severity and subclinical atherosclerosis [7]. Moreover, current evidence suggests that progressive MASLD is an independent risk for developing CVD, particularly in patients with obesity and advanced fibrosis [27]. In this study, our multivariate analysis revealed that a BMI of ≥30, the presence of T2DM, along with the patient’s age, fibrosis severity, and high plasma TMAO levels, were independent factors associated with CAC scores exceeding 100 AU. Indeed, this cut-off value of CAC is considered a high risk for future development of CVD events in a systematic review, indicating that lifestyle modification and therapeutic intervention, such as statins, are required to prevent further complications [28]. Together, these results suggest that the intersection of MASLD severity, metabolic disorders, including obesity and T2DM, and gut-derived metabolites displays a complex clinical scenario challenging healthcare in the management and prevention of CVD.

Obesity and associated metabolic disorders, including T2DM and MASLD, have markedly increased over the last decades. Recent advances suggest that gut microbiota play a crucial role in obesity and its related diseases [29]. Gut dysbiosis, characterized by an altered intestinal microbial balance, is a disruption of microbial composition and function that affects several pathophysiological conditions, including MASLD and CVD [11,14]. In this report, one of the main findings was the changes in gut microbiota, particularly altered beta-diversities but not alpha-diversities. These data suggested that the overall bacterial richness and evenness were comparable between groups according to the BMI of patients; however, the specific gut bacterial genera presented in each group were not the same. Regarding gut microbial community at the genus level, we identified several bacterial genera enriched in the obese population, including *Megamonas*, *Sutterella*, *Catenibacterium*, and *Odoribacter*. In contrast, various SCFA-producing bacteria, such as *Blautia*, *Bifidobacterium*, *Anaerostipes*, and *Butyricicoccus*, had a higher abundance in individuals with lower BMI categories. Aligned with our data, a higher abundance of *Megamonas* has been reported to be associated with the development of obesity and MASLD in Chinese children and adolescents [30]. Similarly, *Megamonas* was shown to be associated with obesity in Mexican populations [31]. Interestingly, a recent large-scale study involving over 1000 Chinese individuals identified potential obesity-related gut microbial enterotypes, specifically the *Megamonas* cluster, which is enriched in obese individuals [32]. In a mouse model and functional analysis, the bacteria in this cluster may contribute to the development of obesity by inhibiting fatty acid transport, degrading myoinositol, and increasing dietary lipid absorption [32]. Collectively, these data indicate that *Megamonas* is a potential obesity-related gut microbiota and establish its clinical importance through human studies, animal models, and cell experiments.

Additionally, a meta-analysis demonstrated that the relative abundances of *Sutterella* and *Catenibacterium* were significantly higher in obese adults compared to non-obese adults, findings consistent with our results [33]. *Sutterella* is a genus of bacteria associated with several human diseases, including inflammatory bowel disease (IBD) [34]. Recent data suggested a potential link between its abundance and obesity, particularly in children and adolescents, associated with high-fat diets and inflammation [35]. Moreover, *Sutterella* was associated with fibrosis in morbidly obese patients with MASLD undergoing bariatric surgery [36]. *Catenibacterium* was shown to be markedly enriched in patients with end-stage renal disease [37]. The bacterium was also found to be increased in the obese population residing in areas with transitions towards Westernized diets and inactive lifestyles [38]. Together, our report and previous data supported a significant distinction in gut microbiota profiles in obese individuals with MASLD. Understanding the specific microbial composition associated with obese MASLD may lead to more personalized and targeted therapy.

Accumulating evidence also suggests that gut dysbiosis plays a role in influencing the development and severity of CVD and SCA. Here, we demonstrated the significant alterations of gut microbial composition in our MASLD cohort according to SCA severity. This study is one of the first to report on the characteristics of gut dysbiosis in patients with MASLD, regarding SCA. The CAC score was positively correlated with the relative abundance of potentially harmful bacteria such as *Ruminococcus gnavus* and *Bacteroides*. In contrast, the CAC score was negatively correlated with the abundance of several SCFA-producing genera, including *Faecalibacterium*, *Agathobacter*, among others. Additionally, in patients with high SCA scores of above 100 AU, enrichment in *Ruminococcus gnavus*, *Bacteroides*, and *Lachnoclostridium*, together with depletion of several SCFA-producing bacteria, including *Faecalibacterium*, *Agathobacter*, *Fusicatenibacter*, *Eubacterium*, *Lachnospiraceae*, and *Christensenella*, was observed compared to those with less severe CAC scores.

Recent data have suggested a crucial role of *Lachnoclostridium* in promoting the development of atherosclerosis via its production of TMA [39]. Other reports have also demonstrated that *Ruminococcus gnavus* is consistently associated with various disorders, such as inflammatory bowel disease (IBD), neurological diseases, malignancies, and cardiometabolic disorders, including MASLD, T2DM, and obesity [40]. Moreover, the enriched abundance of *Ruminococcus gnavus* was also found in patients with CVD after adjusting for conventional risk factors, such as dyslipidemia and T2DM [41,42]. A large population-based cohort also indicated a strong association between the relative abundance of *Ruminococcus gnavus* and the amount of body fat, after adjustment for multiple confounders [43]. In this context, recent data have provided mechanistic insights suggesting that bacterium-derived tryptamine and phenethylamine may play a pathogenic role in connection with cardiometabolic disorders [44].

SCFAs, principally including acetate, propionate, and butyrate, are the main metabolites generated by microbial fermentation of dietary fiber. SCFAs are implicated in mediating various physiological functions, including intestinal epithelial proliferation, gut barrier permeability, energy modulation, and immune response [45]. Increasing interest is being shown in the role of butyrate in cardio-protective effects against atherosclerotic complications. As demonstrated in both in vitro and in vivo studies, butyrate regulates gene expression related to lipid and glucose metabolism, thereby suppressing the oxidative stress of endothelial cells and enhancing vascular integrity, which may reduce the occurrence and progression of atherosclerosis [46]. In a longitudinal study of overweight/obese people, for example, increased serum or fecal butyrate is associated with lowered systolic blood pressure and the prevalence of HT, thereby reducing the risk of CVD [47]. In a large-scale study of Chinese populations, several major SCFA-producing bacteria, including *Roseburia* and *Faecalibacterium*, significantly declined in patients with atherosclerotic CVD compared to healthy controls, indicating a potential protective role of these bacteria and their metabolites in atherosclerosis [41]. Moreover, other cross-sectional studies have confirmed the reduced abundance of SCFA-producing bacteria in patients with subclinical atherosclerosis [48,49]. Similarly, our study demonstrated that several SCFA-producing bacteria were depleted in patients with high CAC scores. Among these bacterial taxa, *Faecalibacterium* has emerged as an independent predictor for CVD occurrence and progression, potentially impacting reduced LPS synthesis and enhanced intestinal mucosal barrier integrity [50].

Growing evidence has revealed that gut dysbiosis plays a detrimental role in the development of atherosclerosis by increasing intestinal permeability and producing injurious metabolites [16]. Indeed, this so-called ‘leaky gut’ phenotype has been demonstrated in several animal and human studies of CVD [14]. Disrupted intestinal permeability facilitates the translocation of microbes and related metabolites into the systemic circulation, which induces the production of proinflammatory mediators, immune responses, and vascular dysfunction, contributing to the development of atherosclerosis [51]. In this context, our study demonstrated that plasma levels of I-FABP, a surrogate marker of intestinal damage, were significantly higher in univariate analysis among patients with CAC ≥ 100 AU than those with CAC < 100 AU. Our report also showed that plasma levels of I-FABP were positively correlated with liver fibrosis measured by MRE and total CAC scores, reflecting the potential role of disrupted intestinal permeability in promoting adverse liver and cardiovascular events. Elevated circulating I-FABP levels were also shown in a previous study demonstrating a positive association with adverse clinical outcomes in patients with CVD [52].

TMAO, a metabolite derived from gut microbiota, originates from TMA, which is produced by consuming dietary precursors such as choline, carnitine, and betaine, abundantly found in meat and eggs [15]. After TMA is generated, it is absorbed and subsequently converted into TMAO by hepatic flavin-containing monooxygenase 3 (FMO3) enzyme before being released into the circulation [15]. Although its exact role in cardiometabolic disorders is not fully clarified, it has been constantly shown that TMAO is directly or indirectly involved in the pathogenesis of CVD, as well as representing a significant risk factor affecting the prognosis of CVD [53]. In this study, elevated circulating TMAO levels were independently associated with increased SCA scores in multivariate analysis, consistent with previous results from a multicenter study in Thailand [54]. Of note, we also demonstrated that TMAO levels had a positive correlation with the abundance of Bacteroides and *Escherichia_Shigella*. In contrast, TMAO exhibited a negative correlation with the abundance of several SCFA-producing bacteria, including Faecalibacterium and Agathobacter, among others. These results indicated that enriched inflammatory bacteria and depleted SCFA-producing genera might play a role in the production of TMAO and be connected to the severity of SCA, as demonstrated in this study.

TMAO is considered an independent risk factor for CVD events in a meta-analysis, and elevated TMAO levels have been related to an increased risk of CVD events, including coronary atherosclerosis and heart failure [55]. For example, the analysis of a large cohort revealed a significant correlation between elevated circulating TMAO levels and increased atherosclerotic risk [56]. Preclinical and clinical data have also suggested mechanistic links between TMAO and the development of various cardiometabolic events, including the promotion of platelet aggregation and the induction of vascular inflammation [16]. To strengthen these findings, the selective targeting of the TMAO signaling pathway via small molecules that directly inhibit microbial-derived TMA production has been shown to improve the clinical consequences of atherosclerosis and vascular thrombosis [57].

Despite the valuable results of this study, some limitations should be acknowledged. First, this study identified associations between gut microbiota and clinical variables in patients with MASLD and SCA, although these findings do not establish a cause-and-effect relationship. The study also included a relatively small sample size of patients with MASLD, which might be susceptible to selection bias; for instance, our data showed a higher average BMI among younger than older individuals, contrary to most reports. Moreover, the report was designed as a cross-sectional study conducted in a single institute in Thailand, and longitudinal cohorts among other populations should further confirm our findings. Additionally, the complexity of the gut microbiota is modulated by several factors, including lifestyle and dietary patterns. Thus, these factors may influence the gut microbial profiling analysis in our cohort, given the limited dietary information available. Finally, the gut microbial composition was characterized by 16S rRNA sequencing, which may be inadequate for providing species-level information and comprehensive functional insights. Accordingly, whole-genome or shotgun metagenomics would offer additional details regarding microbial communities and functional assessment among strain-level differences. Considering these limitations, our strength was the inclusion of a well-characterized cohort of patients with MASLD at various stages of fibrosis. Moreover, our analysis using multiple logistic regression to examine the association with CAC scores allowed us to evaluate multiple factors and minimize their confounding effects.

## 5. Conclusions

In conclusion, our study provided significant evidence of gut dysbiosis and metabolites, particularly TMAO, in association with not only obesity but also SCA severity in patients with MASLD. The altered gut microbiota in patients with a high SCA burden was characterized by an increased abundance of pathogenic bacterial taxa, particularly *Ruminococcus gnavus*, and a reduction in SCFA-producing genera, such as *Faecalibacterium*, among others. These data lead to a better understanding of the mechanistic role of gut dysbiosis concerning obese MASLD and SCA. Given the growing evidence linking TMAO to CVD, the role of this metabolite as a complementary biomarker in identifying high-risk individuals within the MASLD population warrants further investigation. Additionally, our results may support personalized interventions, such as modulating gut dysbiosis through probiotic or prebiotic supplements, which could be beneficial in slowing CVD progression in patients with MASLD [58].

## Figures and Tables

**Table 1:** Characteristics of patients with MASLD according to BMI.

Characteristics	Low BMI (<30 kg/m^2^) (*n* = 151)	High BMI (≥30 kg/m^2^) (*n* = 51)	*p*-Value
Age (years)	58.8 ± 12.7	51.4 ± 13.9	0.001 *
Gender Male Female	80 (53.0) 71 (47.0)	24 (47.1) 27 (52.9)	0.284
BMI (kg/m^2^)	25.9 ± 2.2	33.7 ± 4.8	<0.001 *
Presence of type 2 diabetes	99 (65.6)	33 (64.7)	1.000
Presence of hypertension	90 (59.6)	24 (47.1)	0.142
Presence of dyslipidemia	98 (64.9)	33 (64.7)	1.000
Statin use	62 (41.1)	23 (45.1)	0.626
Smoking	11 (7.3)	6 (11.6)	0.447
Hemoglobin (g/dL)	13.8 ± 1.6	14.0 ± 1.9	0.636
White blood count (10^3^/µL)	6.5 ± 1.9	7.7 ± 2.1	0.011 *
Platelet count (10^3^/µL)	241.5 ± 71.9	270.3 ± 70.1	0.019 *
Serum creatinine (mg/dL)	0.9 ± 1.0	1.0 ± 1.6	0.552
Estimated glomerular filtration rate (eGFR) (mL/min/1.73 m^2^)	91.2 ± 20.7	96.0 ± 25.0	0.189
Total bilirubin (mg/dL)	0.8 ± 0.3	0.7 ± 0.3	0.846
Serum albumin (g/dL)	4.3 ± 0.3	4.4 ± 0.4	0.235
Aspartate aminotransferase (IU/L)	28.8 ± 14.6	27.2 ± 10.3	0.469
Alanine aminotransferase (IU/L)	35.5 ± 23.1	38.6 ± 19.6	0.390
Alkaline phosphatase (IU/L)	74.7 ± 27.5	68.2 ± 16.3	0.286
Magnetic resonance elastography (kPa)	2.9 ± 1.2	2.7 ± 1.1	0.349
Proton density fat fraction (%)	11.2 ± 7.5	15.0 ± 7.1	0.002 *
Coronary artery calcification (AU)	204.0 ± 378.6	450.3 ± 511.5	0.015 *

Data expressed as mean ± SD or *n* (%), * *p*-value < 0.05.

**Table 2:** Factors associated with high CAC scores (≥100 AU).

Factors	Category	Univariate Analysis		Multivariate Analysis	
		OR (95% CI)	*p*-Value	OR (95% CI)	*p*-Value
Age (years)	≥60 vs. <60	4.27 (1.83–9.94)	0.001 *	9.20 (2.30–36.87)	0.002 *
Gender	Male vs. Female	0.85 (0.38–1.88)	0.686		
BMI (kg/m^2^)	≥30 vs. <30	2.45 (1.05–5.71)	0.038 *	5.07 (1.41–18.21)	0.013 *
Diabetes	Yes vs. No	4.41 (1.81–10.79)	0.001 *	3.89 (1.20–12.58)	0.023 *
Hypertension	Yes vs. No	4.31 (1.84–10.07)	0.001 *	0.78 (0.20–2.98)	0.716
Dyslipidemia	Yes vs. No	1.58 (0.68–3.69)	0.287		
Smoking	Yes vs. No	2.24 (0.68–7.36)	0.184		
Aspartate aminotransferase (IU/L)	≥40 vs. <40	2.28 (0.78–6.69)	0.134		
Alanine aminotransferase (IU/L)	≥40 vs. <40	0.80 (0.34–1.88)	0.612		
Albumin (g/dL)	<4.0 vs. ≥4.0	2.82 (0.28–28.56)	0.379		
Platelet count (10^9^/L)	<150 vs. ≥150	3.04 (0.88–10.49)	0.078		
eGFR (mL/min/1.73 m^2^)	<90 vs. ≥90	2.18 (0.92–5.18)	0.077		
Liver steatosis grade	S2-S3 vs. S1	0.70(0.30–1.61)	0.397		
Liver fibrosis stage	F3-F4 vs. F0-F2	5.38 (1.93–14.94)	0.001 *	5.30 (1.37–20.56)	0.016 *
Fecal BCoAT level	Low vs. High	2.30 (1.02–5.17)	0.044 *	1.19 (0.40–3.59)	0.752
Plasma I-FABP (ng/mL)	≥1000 vs. <1000	3.39 (1.30–8.83)	0.012 *	1.35 (0.32–5.60)	0.683
Plasma LBP (ng/mL)	≥15,000 vs. <15,000	1.34 (0.60–3.00)	0.470		
Plasma choline (µM)	≥40 vs. <40	0.96 (0.43–2.13)	0.917		
Plasma betaine (µM)	≥40 vs. <40	1.35 (0.59–3.06)	0.480		
Plasma TMAO (µM)	≥5.0 vs. <5.0	7.76 (2.41–25.01)	0.001 *	5.26 (1.20–23.97)	0.032 *

Data expressed as odds ratio (OR) and 95% confidence intervals (CI); * *p*-value < 0.05.

## Data Availability

The data supporting the findings of this study are available from the corresponding author upon reasonable request.

## Supplementary Materials

The following supporting information can be downloaded at https://www.mdpi.com/article/10.3390/nu17172759/s1, Figure S1: Alpha diversities between the low and high CAC score groups. Figure S2: The principal coordinate analysis (PCoA) of beta-diversity profiling between the low and high CAC score groups. Table S1: Top 20 of relative bacterial composition at the genus level according to BMI groups.
