# Supplementary material for: Gut Dysbiosis and Plasma Trimethylamine Oxide Are Associated with Subclinical Coronary Atherosclerosis in Obese Patients with Metabolic Dysfunction-Associated Steatotic Liver Disease
Source: Nutrients. 2025 Aug 26;17(17):2759. doi: 10.3390/nu17172759 (PMC12430641; doi:10.3390/nu17172759)
Supplement: Supplementary file 1 [file nutrients-17-02759-s001.zip › nutrients-3802385-supplementary.pdf]

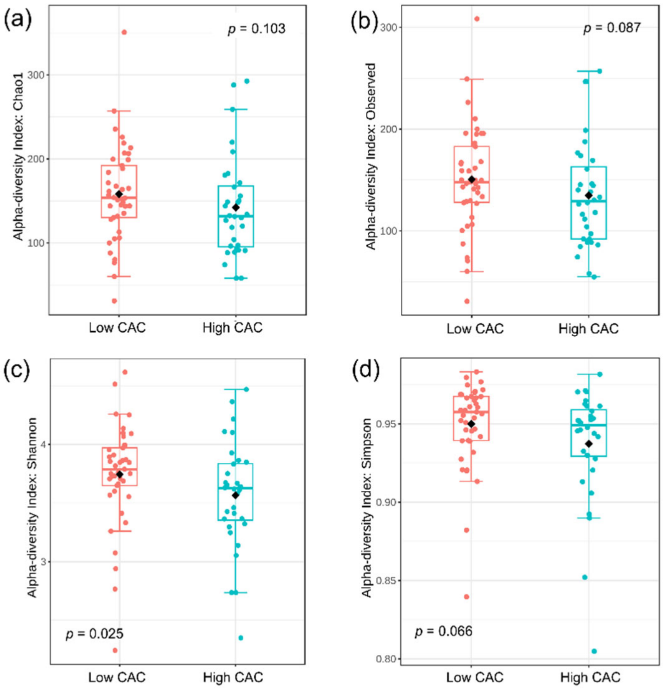


**Figure S1.** Alpha diversities between the low and high CAC score groups: (a) Chao1 index, (b) Observed index, (c) Shannon index, and (d) Simpson index. Statistical measures were median, interquartile range, and outliers. The statistical analysis was performed using the Mann-Whitney test.

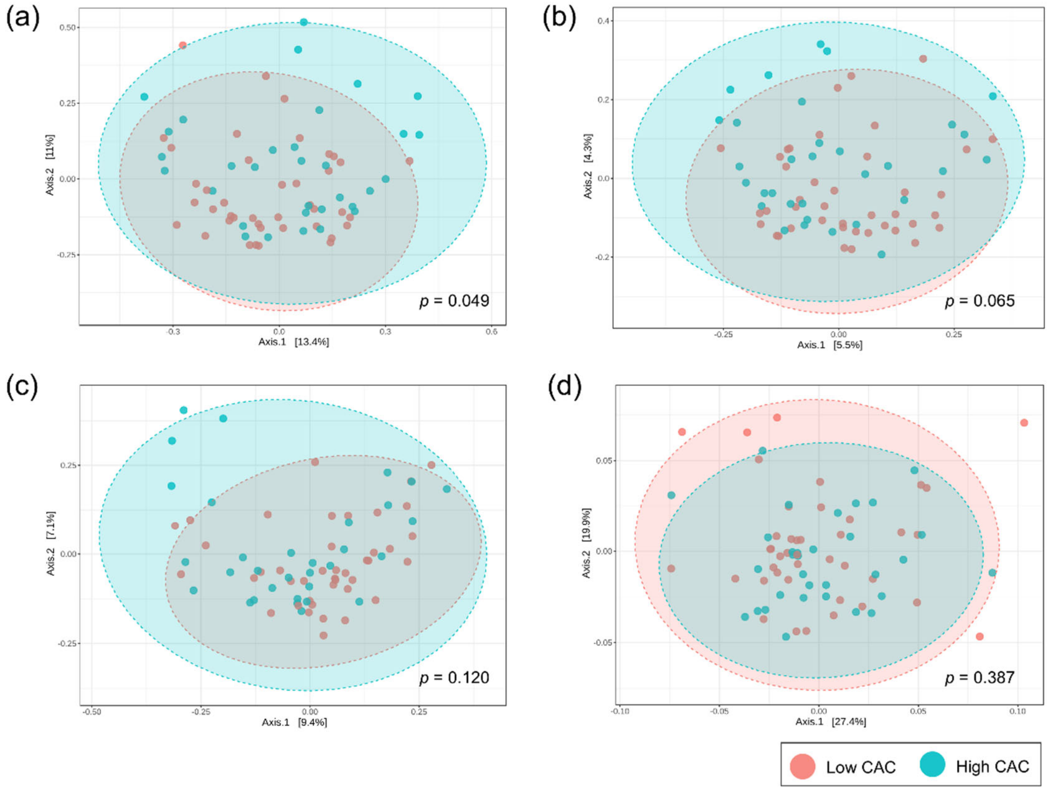


**Figure S2.** The principal coordinate analysis (PCoA) of beta-diversity profiling between the low and high CAC score groups: (a) Bray-Curtis index, (b) Jaccard index, (c) Unweighted Unifrac index, (d) Weighted Unifrac index. The differences in beta diversity were tested by PERMANOVA.

**Table S1.** Top 20 of relative bacterial composition at the genus level according to BMI groups

| **Bacterial genera** | **Low BMI (<30 kg/m²)** | **High BMI (≥30kg/m²)** |
|---|---|---|
| Others | 0.2564 | 0.2495 |
| *g__Bacteroides* | 0.1769 | 0.1564 |
| *g__Blautia* | 0.0956 | 0.0791 |
| *g__Prevotella* | 0.0825 | 0.1115 |
| *g__Faecalibacterium* | 0.0522 | 0.0531 |
| *Not_Assigned* | 0.0393 | 0.0422 |
| *g__Megamonas* | 0.0335 | 0.0555 |
| *g__Bifidobacterium* | 0.0320 | 0.0204 |
| *g__Collinsella* | 0.0319 | 0.0420 |
| *g__Agathobacter* | 0.0220 | 0.0233 |
| *g__Fusicatenibacter* | 0.0220 | 0.0150 |
| *g__Escherichia_Shigella* | 0.0217 | 0.0124 |
| *g__Roseburia* | 0.0193 | 0.0234 |
| *g__Ruminococcus_torques_group* | 0.0183 | 0.0195 |
| *g__Phascolarctobacterium* | 0.0171 | 0.0181 |
| *g__Anaerostipes* | 0.0160 | 0.0074 |
| *g__Dorea* | 0.0135 | 0.0145 |
| *g__Fusobacterium* | 0.0135 | 0.0147 |
| *g__Subdoligranulum* | 0.0127 | 0.0142 |
| *g__Eubacterium_hallii_group* | 0.0123 | 0.0115 |
| *g__Ruminococcus_gnavus_group* | 0.0112 | 0.0164 |
